# Institutionalizing health technology assessment in Egypt: Situational analysis and roadmap

**DOI:** 10.3389/fphar.2022.1014658

**Published:** 2022-11-09

**Authors:** Pilar Pinilla-Dominguez, Shorouk Taha, Hugh McGuire, Ahmed Elagamy, Amal Sedrak, Mary Gamal, Mariam Eldebeiky, Dalia Dawoud

**Affiliations:** ^1^ NICE International—National Institute for Health and Care Excellence (NICE), London, United Kingdom; ^2^ Department of Quantitative Methods for Economics and Management, University of Las Palmas de Gran Canaria, Las Palmas, Spain; ^3^ Central Administration of Health Technology Management (CAHTM), Egyptian Authority for Unified Procurement, Medical Supply and Technology Management (UPA), Cairo, Egypt; ^4^ Faculty of Medicine, Department of Public Health, Cairo University, Cairo, Egypt; ^5^ NICE Science, Policy & Research, National Institute for Health and Care Excellence, London, United Kingdom; ^6^ Faculty of Pharmacy, Cairo University, Cairo, Egypt

**Keywords:** health technology assessment (HTA), Egypt, situational analysis, egyptian authority for unified procurement, medical supply and the management of medical technology (UPA), universal health coverage (UHC)

## Abstract

**Objective:** To conduct a situational analysis with the aim to inform future health technology assessment efforts (HTA) in Egypt.

**Introduction:** The Egyptian government has set universal health coverage as a 2030 target. Several agencies have been created in the context of the ongoing healthcare reform. The Egyptian Authority for Unified Procurement, Medical Supply and the Management of Medical Technology (UPA) is one of them and was established to support strategic procurement using HTA.

**Methods:** Description of the development of HTA in Egypt supported by a literature search as part of a scoping exercise, and a stakeholder analysis and identification of HTA capacity survey, based on previous surveys, with relevant stakeholders conducted in 2022. This was followed by a stakeholder event where results were shared and further contextualized.

**Results:** The UPA is expected to evaluate the cost-effectiveness of health technologies and public health programs. The HTA process is being developed, focusing on the assessment of the value of new pharmaceuticals being introduced to the Egyptian market. A total of 16 participants responded on behalf of their organizations to the stakeholder analysis and identification of HTA capacity survey. More than 80% of the respondents were familiar with current efforts conducted by UPA and strongly support the implementation of HTA in Egypt. Transparency was highlighted as an important criterion. Over 90% of the respondents mentioned economic analyses as an HTA product being developed in Egypt, and medicines were the type of technology that stakeholders ranked as first in the rank of health technologies that need the output from HTA urgently. Capability building and training were highlighted as areas in which further support is required.

**Conclusion:** This study represents the first attempt to describe the current path for HTA in Egypt. There seems to be momentum in Egypt to proceed and advance with HTA institutionalization. It would be important that next steps are built on the skills and capabilities already in place in Egypt, ensure methods and processes are in place and up to date and involve the wider system in Egypt so stakeholders can appropriately contribute and participate in the HTA process.

## Introduction

The healthcare system in Egypt is complex and fragmented, made up of different public and private providers and funding bodies (Mathauer et al., 2019). Currently the healthcare system in Egypt is constrained by an absence of universal coverage and equitable access to healthcare services across the country. Low central government investment in health has been reflected in an increased reliance on the private sector for the provision of health services and resulted in out-of-pocket expenditure accounting for around 72% of total health spending (Ministry of Health and Population, 2014). In 2010, it was reported that Egypt spent 4.7% of GDP on health (Gericke and Busse, 2018).

In 2009, Egypt adopted a new pricing method based on the lowest retail price in any country of the world (external reference pricing), but this has led to both price increases and decreases without substantive implications on affordability (Mohamed and Kreling, 2016). Calls have been made to encourage countries in the Middle East and North Africa (MENA) region, including Egypt, to move forward in their pricing policies beyond external reference pricing, and introduce value assessment mechanisms (Kanavos et al., 2020). Beyond Tunisia, that has the Tunisian Authority of Assessment and Accreditation in Healthcare (INEAS), Egypt and the Kingdom of Saudi Arabia seem to be amongst the first countries in the region to publicly announce and commit to the incorporation of value assessment mechanisms, such as health technology assessment (HTA) as part of their healthcare reforms (Fasseeh et al., 2020; Kanavos et al., 2020).

The use of HTA is seen as an important mean for ensuring the sustainability of a universal health coverage (UHC) system (Chalkidou et al., 2016). HTA is an example of an output of explicit, deliberative priority-setting processes and is defined as a multidisciplinary process that uses explicit methods to determine the value of a health technology at different points in its lifecycle. The purpose is to inform decision-making in order to promote an equitable, efficient, and high-quality health systems (O’Rourke et al., 2020).

The Egyptian government has set UHC as a target to be achieved by 2030 *via* the Universal Health Insurance (UHI) System as outlined in the Universal Health Insurance Law No. 2/2018. This law describes plans to restructure the healthcare system by making healthcare services affordable to all citizens with those in vulnerable groups receiving subsidies from the government. The UHI system is based on the principles of social solidarity and includes a separation of functions between the provider and the purchaser of services. (Mathauer et al., 2019; World Bank Group, 2020)

Law No. 2/2018 also envisioned the creation of new bodies: the Universal Health Insurance Agency (UHIA), the General Authority of Health Care (GAHC), and the General Authority for Healthcare Accreditation and Regulation (GAHAR). The UHIA will act as the purchaser of services and be responsible for pooling provider payments, and the management and investment of the UHI fund. The UHIA will also be able to purchase health services for private insurance beneficiaries under special arrangements with private insurers. The GAHC will act as the public provider of primary, secondary, and tertiary level healthcare services, report to the Ministry of Health and Population (MOHP) and have ownership of the public health facilities to facilitate economies of scale, efficiencies and optimize integration of care across the system. The GAHAR will be the regulator and accreditor providing standards on structural quality, clinical processes, and patient outcomes.

The Egyptian Authority for Unified Procurement, Medical Supply and the Management of Medical Technology (UPA) was subsequently created by Law No. 151/2019. The UPA was established to support the efforts to achieve UHC by fostering strategic procurement. The UPA will procure and manage the supply of pharmaceuticals, medical equipment, and other medical supplies for all public healthcare entities. The UPA is expected to procure health technologies in a cost-effective manner and be able to negotiate prices for health technologies for both public and private sector providers. Furthermore, the Egyptian Drug Authority (EDA), which was also established by Law No. 151/2019 as a public service authority, was created to act as the regulatory authority responsible solely for the registration, licensing, inspection and supervision of all pharmaceutical, cosmetic products and medical equipment. They also have responsibility for setting public prices for new technologies. (World Bank Group. 2020).

The Universal Health Insurance Law No. 2/2018 sets out the legal framework for how the different agencies will work in a coordinated manner with the UHIA and GAHC. GAHC facilities will be contracted by the UHIA only after GAHAR accreditation has been obtained. HTA will be the responsibility of the UPA, who is expected to evaluate the cost-effectiveness of health technologies and public health programs. The UPA will only undertake HTA of technologies that have previously received regulatory approval by the EDA. (World Bank Group. 2020).

In order to institutionalize HTA, countries need to navigate different elements of dynamic policy making. Castro et al. developed a framework for institutionalizing HTA that starts from a policy setting stage, in which systematic priority setting is described as the key policy to develop (Castro and Suharlim, 2020). This leads into an agenda setting stage, where the window of opportunity is sought and policy analyses to formulate HTA policies are conducted. The next phase is the policy formulation stage, which includes benchmarking good practices and conducting situational analyses and stakeholder exercises. This is followed by the adoption stage, where legal frameworks, institutional arrangements and regulation are established, and capacity is assessed. This then leads to the implementation stage, with the development of methodological guidelines and process guides, alongside capability building efforts. Importantly, this also needs to be followed by an impact evaluation stage, where measures of impact and key performance indicators are set up and data collection is put in place to generate such insights. This will in turn create a feedback mechanism to keep modifying HTA policies according to learnings gathered.

Following this framework, this situational analysis aims to inform future HTA efforts in Egypt, particularly as it moves from the agenda setting/policy formulation stage to adoption and implementation. It includes an overview of the development of HTA in Egypt, stakeholder analysis and assessment of capacity and support. It allows for getting deeper understanding of opportunities, challenges, and next steps for implementing HTA in Egypt. The situational analysis is considered a crucial step when establishing a HTA policy in a country (Jeffery et al., 2018; Castro and Suharlim, 2020; Bertram et al., 2021). Health system context plays a major role facilitating the implementation of HTA policy, and stakeholder involvement is an essential aspect to guarantee buy-in and legitimacy of HTA processes.

## Methods

The situational analysis was co-developed and co-drafted by the UPA and the National Institute for Health and Care Excellence (NICE) International and was informed by the following activities.

### Literature search to inform the context of HTA in Egypt and scoping exercise

We conducted a literature search for published and grey literature to identify information related to HTA in the Egyptian context. This included searches of Medline and EMBASE as well as grey literature including websites from public authorities in Egypt and HTA networks such as the International Society for Pharmacoeconomics and Outcomes Research (ISPOR) Egypt regional chapter. For the Medline search Medical Subject Headings (MeSH) and text-word searches were used to identify literature of interest (see supplementary Table S1). The search was focused on countries in the MENA region. A translated search was run in EMBASE. The search was performed in August 2021 and was limited to January 2011 onwards in order to ensure that only current publications on HTA were reviewed.

The literature search identified 2,220 results of which 2,206 were excluded on title and abstract screened as not relevant to the HTA context in Egypt. The full text of the remaining 14 publications was retrieved. Information gleaned from the literature searches was supplemented with documents and information prepared and shared verbally and in written format by the UPA detailing the context of the Egyptian healthcare system, upcoming changes and current process and expectations for HTA in Egypt during the scoping exercise. The UPA described the ongoing development of the HTA process in Egypt. NICE International commented in this process, which is described in the results section of this manuscript. The results and learnings were shared between the UPA and NICE International during a scoping meeting, where the approach to HTA from both UPA and NICE were shared and compared.

### Stakeholder analysis and identification of HTA capacity survey

In 2022 a survey was delivered to targeted stakeholders in Egypt to assess the awareness and perceptions of stakeholders of the current HTA system and institutional aspects in Egypt, stakeholders’ attitudes towards HTA in Egypt and their expectations, as well as current HTA capacity and needs in Egypt. The survey was based on previous surveys used to identify stakeholders and HTA capacity (Schmeer, 2000; Li, 2017; Downey et al., 2018; Vlad, 2018; Hollingworth et al., 2021). These surveys have been used to conduct situational analyses in other countries (Addo et al., 2020; Downey et al., 2020; Sharma et al., 2020; Elfarra, 2021). The survey was slightly adapted and modified by the UPA and NICE International for the purpose of this project and context in Egypt. The survey included open and closed questions, including Likert scales. A copy of the full survey can be found in the supplementary materials.

The organizations invited to participate in the survey followed a convenience sample approach based on a prioritized list of organizations involved in the current UPA’s HTA workflow (*n* = 16). These were organizations that were expected to be part of or engage directly with the HTA process in Egypt. The list of stakeholders was prepared and prioritized guided by a stakeholder checklist for priority setting in low and middle-income countries (Vlad, 2018). This list included national government organizations (*n* = 6), life sciences industry (*n* = 2), public hospital (*n* = 1), consultancy companies (*n* = 2), HTA networks with presence/relationship with Egypt and MENA region (*n* = 1), academia (*n* = 2), and international organizations including the World Bank and the WHO office in Egypt (*n* = 2). Official invitations were sent to the prioritized stakeholders to nominate a representative to participate in answering the survey. Respondents were at director, president/vicepresident, senior specialist and professor level. Participants were asked to sign consent forms for participation which were collected before the survey was sent. Subsequent consent was requested to publish the aggregated results. An awareness event was held by the UPA to explain the project and objective of the survey with all invited organizations, which was attended by all of them (*n* = 16). It was also an opportunity to provide an overview of the questions to ensure they were understandable by participants. The survey was distributed online, and participants were given 3 days after the awareness event to complete it.

UPA proposed using a general descriptive analysis and inductive qualitative approach, identifying key themes using Excel for sorting data and categorizing it. Answers to open questions were categorized for this analysis based on common responses and summarized using descriptive statistics. NICE International reviewed the responses, categorization and analyses. Figures were developed using Datawrapper.

In April 2022, the UPA and NICE International held a stakeholder event to share the results and findings of the stakeholder analysis and HTA capacity survey. All the organizations that took part in the survey were invited to an event in Cairo and 75% of them attended. The invitation was extended to further pharmacy and medical academic institutions in Egypt and one further organization attended. At the event both the UPA and NICE International presented the results and had a moderated discussion.

## Results

### HTA agenda setting: Development of the HTA process and methods in Egypt

Based on the findings from the scoping exercise, the HTA process is currently being developed by the UPA, focusing at this stage on the assessment of the value of new pharmaceuticals being introduced to the Egyptian market. The objective of this HTA is to ensure that the assessment is conducted to support reimbursement decisions and to add the technology to the list for procurement.

The draft HTA process developed by the UPA is expected to last approximately 3 months. The UHIA will work with the UPA to identify priority disease areas. At this stage, the process is restricted to the evaluation of either ‘first to market’ pharmaceuticals that have a high impact on the budget or innovative products. In the future, a HTA evaluation could be requested by different stakeholders. The UPA is finalizing a pharmaceutical value dossier submission template for companies to complete. During the assessment stage, there will be an initial evaluation of the submission conducted by UPA staff including the economic and clinical studies. Once the UHIA becomes the single payer, the UHIA and the UPA will collaborate in the HTA process. At this point the UPA will assess the cost effectiveness of the health technologies and programs and the UHIA will assess the budget impact based on the population covered by the UHIA. The HTA recommendations resulting from the appraisal of the value dossier will be advisory and will inform the pricing negotiations for procurement and reimbursement purposes.

The evaluation is then reviewed by a scientific committee. This scientific committee includes methodologists in clinical research, health economists and clinical topic experts. Once the scientific committee has reached a recommendation, the UPA then informs the company if the submission is accepted or rejected. After the assessment and appraisal of the evidence, the UPA evaluation will be submitted to the recommendation committee who will make decisions on the appropriate price for the technology. This committee includes representatives of the UHIA, MOHP, hospitals and other entities entitled by law 151/2019 and the procurement central administration team at the UPA. The recommendation committee will also consider the assessment of the budget impact analysis which will feed into any further negotiations and agreement on the final price.

The former Central Administration for Pharmaceutical Affairs (CAPA), a regulatory agency within the MOHP, developed scientific guidance on how to conduct and report a pharmacoeconomic study in 2013 (Elsisi et al., 2013). This guidance covers a reference case for Egypt. This includes the perspective, indication, choice of comparator, target population, subgroup analysis, preferred analytical technique, time horizon, choice of outcome measure, preferred method to derive utility, synthesis of clinical and economic evidence, costs to be included, sources of costs, modelling, discounting costs and outcomes, uncertainty, equity issues, generalizability and presenting results. CAPA is now part of the EDA. Following the reform, HTA responsibilities, including methodology sit within the UPA. UPA is currently developing a HTA methods guide for the evaluations.

### Policy formulation: Stakeholder and HTA capacity analysis

#### Organizations with a central role in HTA in Egypt

The majority of the respondents (14 out of 16; 88%) highlighted that UPA has a central role in HTA in Egypt (see Table 1). Out of the 14 respondents who mentioned the UPA as having a central role in HTA, the majority considered their role as mandatory, which contradicts the intention of the UPA process to be advisory, highlighting a mismatch in expectations. In terms of stakeholders’ perceptions about other organizations having a central role in HTA, the UPA was followed by other government authorities such as the MOHP, the UHIA and the EDA, each of them being mentioned by 56% of the respondents showing that the majority of the respondents were familiar with the relevant government organizations aimed to take part in the HTA process being introduced as a result of the ongoing healthcare reform.

**TABLE 1:** Stakeholders’ perceptions about the organizations with a central role in HTA in Egypt.

Respondents N = 16	Q1: In Egypt, are you aware of any central organisation(s) undertaking the role of HTA? If yes, please state their name. You may select more than one optionQ2: What is the current status of recommendations from the organisation(s) named in response to question 1?			

	**MOPH**	**Advisory**	**Mandatory**	**Other**
**n**	9	6	1	2
**%**	56%	67%	11%	22%
	**EDA**			
**n**	9	6	2	1
**%**	56%	67%	22%	11%
	**UHIA**			
**n**	9	5	3	1
**%**	56%	56%	33%	11%
	**UPA**			
**n**	14	4	8	2
**%**	88%	29%	57%	14%
	**NGO**			
**n**	3	2	0	1
**%**	19%	67%	0%	33%
	**Private sector**			
**n**	5	4	0	1
**%**	31%	80%	0%	20%
	**Other**			
**n**	4	3	1	0
**%**	25%	75%	25%	0%

Abbreviations: HTA: health technology assessment, MOPH: ministry of public health, EDA: egyptian drug authority, UHIA: universal health insurance agency, UPA: unified procurement authority, NGO: Nong-Governmental Organization.

#### Perceptions on the HTA process in Egypt

**FIGURE 1:**
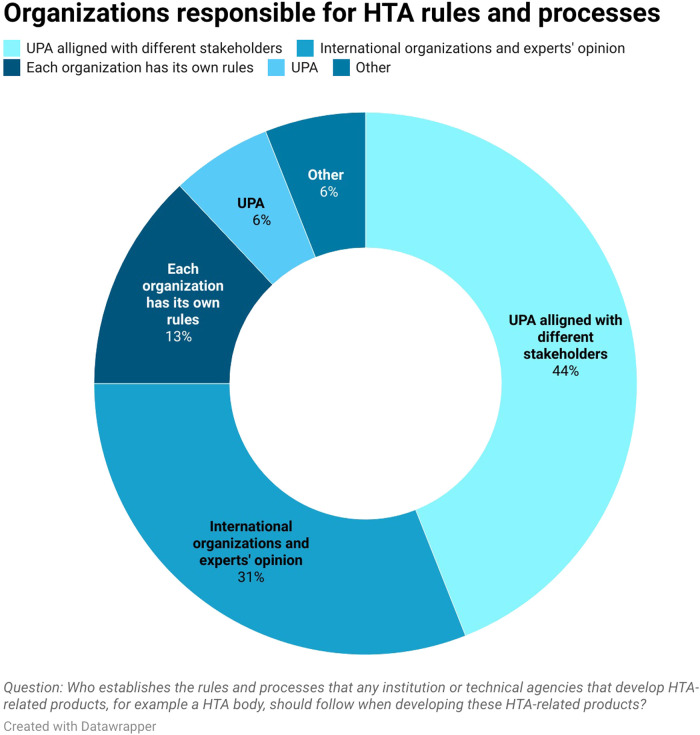
Organizations responsible for HTA rules and processes.

About 38% (6 out of 16) of respondents mentioned that there is no formal process for deciding what HTA-related products will be developed. However, these is a level of familiarity with the ongoing process. About 19% (3 out of 16) of respondents believe that there is a process currently being developed by UPA and UHIA, 25% (4 out of 16) mentioned that UPA, UHIA, and EDA already have a formal process, 13% (2 out of 16) said there is a process only for specific technologies like orphan drugs and oncology medicines and 6% (1 out of 16) of respondents reported that they were unaware. As for who is responsible for establishing HTA rules and processes that the technical agencies should follow when developing HTA products, 44% (7 out of 16) of the respondents noted that it is the UPA aligned with different stakeholders and 6% (1 out of 16) considered the UPA to be sole responsible organization (see Figure 1). 31% of respondents (5 out of 16) mentioned that technical agencies follow international guidelines and HTA expert opinion when developing HTA related products. Among the international guidelines being mentioned, these included guidelines from the WHO, NICE and ISPOR. This shows the stakeholders have some understanding of the methodological developments conducted by the UPA to conduct HTA.

#### HTA products developed in Egypt

**FIGURE 2:**
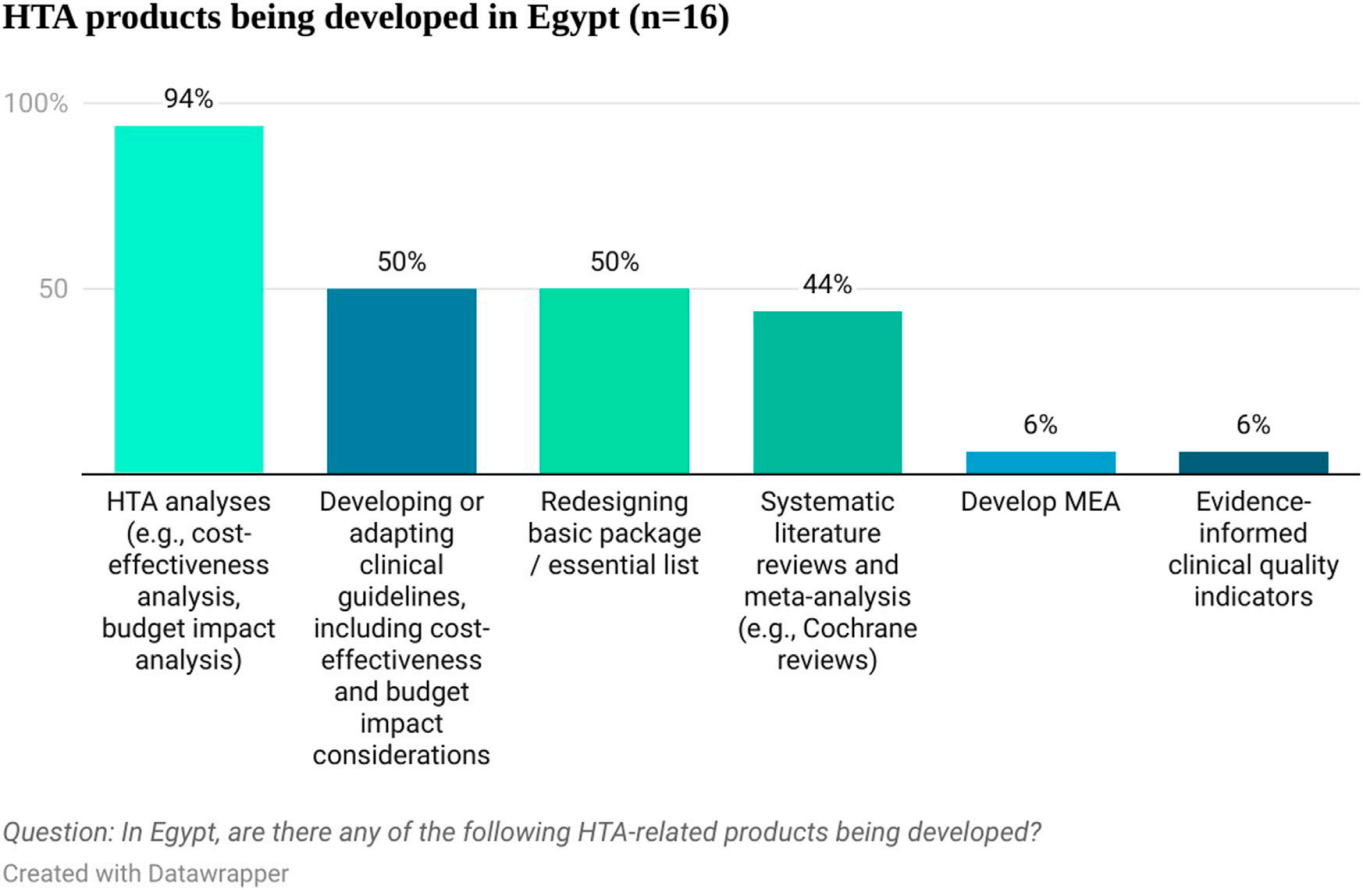
HTA-related products being developed in Egypt.

When asked about different HTA products being developed in Egypt, economic HTA analyses (e.g., cost-effectiveness analysis, budget impact analysis) were mentioned by 94% (15 out of 16) of the respondents. Other products currently being developed in Egypt included developing or adapting clinical guidelines, including cost-effectiveness and budget impact considerations, redesigning the basic package or essential list, systematic literature reviews and meta-analyses, evidence-informed quality indicators and managed entry agreements (see Figure 2). This shows that there is already familiarity with HTA principles in Egypt and that some products are being developed that could inform HTA efforts, including evidence submissions. Respondents considered that those products are produced mainly by governmental bodies (UPA, UHIA, and EDA) and consultancy companies and clinical research organizations. The pharmaceutical and devices industry is thought to be the major payer for the HTA related products development (85.7%, 12 out of 14 respondents). This signals that pharmaceutical companies have the relevant resources to contribute to the process by developing company’s submissions for assessment at a national level. Most respondents (63%, 10 out of 16) considered that Egypt has influence in health policy decisions in similar countries and was mentioned as a reference country for countries in the MENA region, highlighting the relevance of the ongoing HTA efforts in Egypt for the wider MENA region.

#### Stakeholders’ expectations from HTA

**FIGURE 3:**
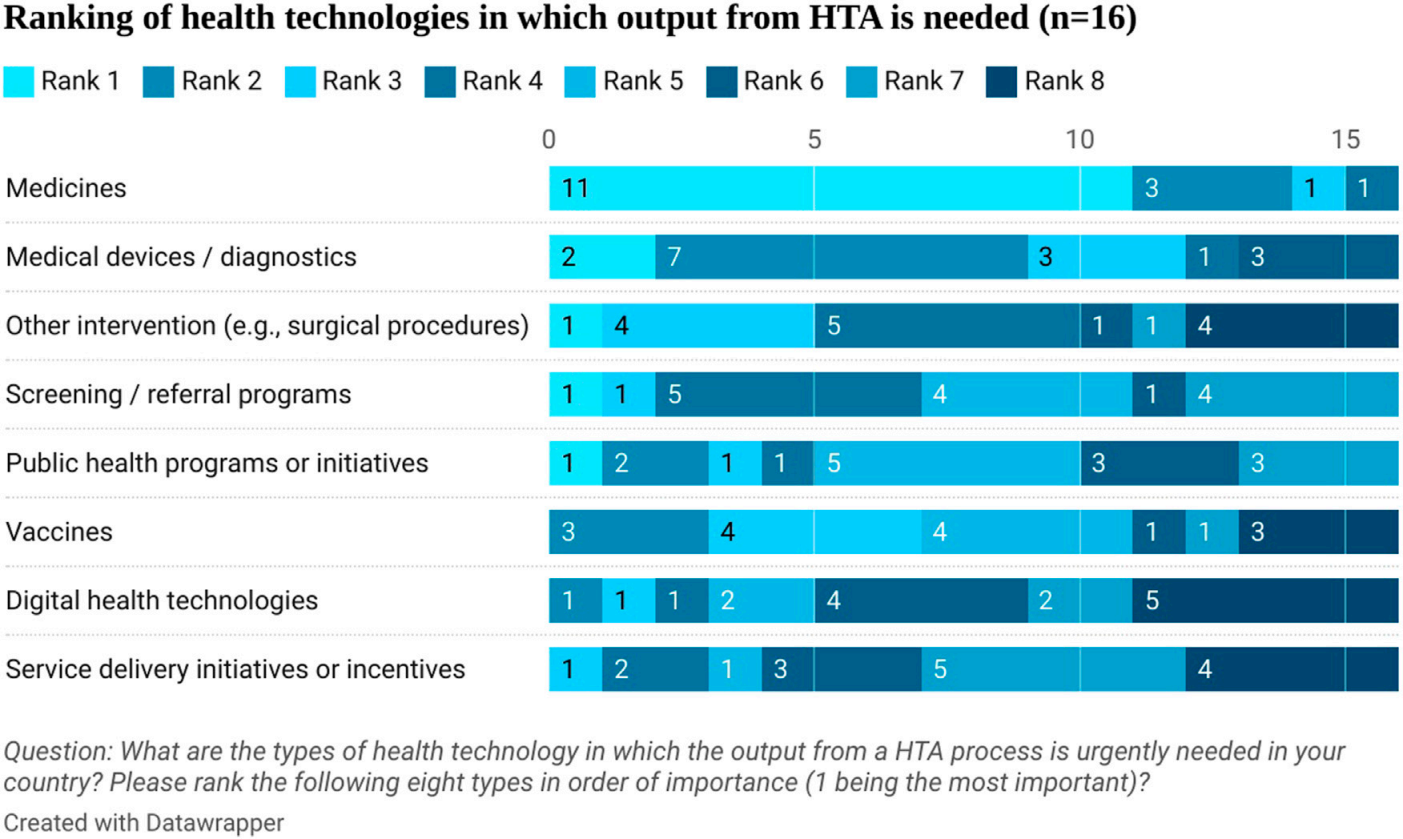
Ranking of health technologies in which output from HTA is needed.

**FIGURE 4:**
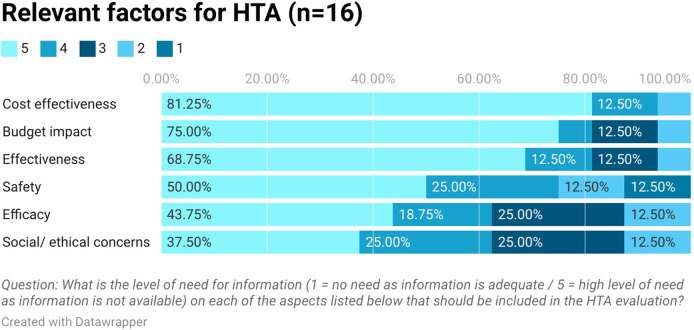
Relevant factors for HTA.

Stakeholders ranked in order the type of health technologies that need the output from HTA urgently. Medicines were first in the rank followed by medical devices and diagnostics (see Figure 3). They highlighted that safety and efficacy information is available from clinical trials but there is a lack of cost-effectiveness, budget impact, and effectiveness information in Egypt (see Figure 4). This aligns with the proposed process being developed by the UPA which will focus first on assessing the clinical and cost-effectiveness of new pharmaceuticals with high budget impact or innovative products.

#### Stakeholders’ attitude towards HTA

Of the respondents, 87% (14 out of 16) strongly support the implementation of HTA in Egypt. The other 13% (2 out of 16) who are somewhat supporting HTA expressed that some conditions need to be present for them to express their support including:

1) Transparency of HTA process
2) The ability to influence decision making
3) HTA process and decisions based on evidence
4) Fast HTA process
5) Support patients and increase patient access to innovative technologies
6) Availability of adequate human and financial resources.

Respondents noted that they will support the implementation of HTA from units within their organizations and by providing essential data for HTA purposes. This shows the actual commitment of stakeholders to actively contribute to the HTA process.

#### HTA capacity in Egypt

**FIGURE 5:**
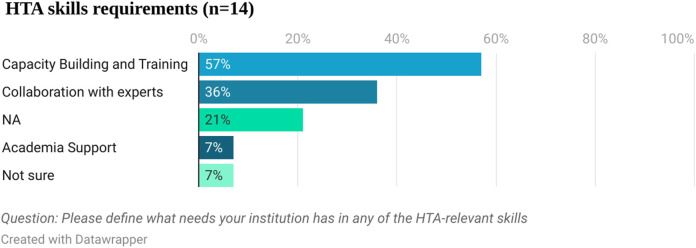
HTA skills requirements.

Fourteen respondents answered the question about the specific HTA relevant skills they have technical needs for (see Figure 5). Capacity building and training was mentioned by 57% (8 out of 14) of the respondents, followed by collaboration with experts (36%, five out of 14).

The capacity for HTA within the UPA comprises members of staff with diploma or masters level qualifications in health economics alongside other qualifications that will support HTA.

Responses to the detailed questions about HTA capacity amongst stakeholders in Egypt were too vague to allow for a formal analysis so a narrative summary of the responses is provided below.

Fewer than 10 staff with a qualification in health economics/health econometrics (first degree, masters, or PhD level), and five or fewer staff with a qualification in epidemiology/biostatistics (first degree, masters, or PhD level) were mentioned by each of the respondents. Respondents mentioned having more staff with medical or pharmacy degrees. Amongst the HTA products published by stakeholders, these included cost-effectiveness analyses, systematic reviews and budget impact analyses. This signals that further efforts should be dedicated to build capacity and capability amongst the wider stakeholders that will be involved in the HTA process.

### Stakeholder event

The results presented resonated with the perceptions on HTA from the attendees to the stakeholder event. They all were aware of the efforts from the UPA and showed willingness to collaborate further, based on their roles. Consultancies and academics highlighted their ability to generate evidence relevant for HTA purposes such as burden of illness, costing studies or economic models.

The attendees also highlighted the importance of having a transparent process, in which each stakeholder can understand how they can contribute and participate in the HTA process. UPA noted their work on this matter and explained their willingness to make this process public once it has been finalized. Involvement of stakeholders before this is finalized is encouraged so they can input into the process. This will contribute to improve inclusiveness and buy-in from the community.

The attendees also encouraged the UPA to publish the HTA reports being conducted in the HTA unit and noted that capability building efforts should currently focus on critical appraisal of economic models. The UPA explained that the HTA reports conducted to date have been part of a pilot and that there are further activities planned in terms of reviews before these are made public and noted that making HTA reports publicly available is within their plans for the future.

## Discussion

This study represents the first attempt to describe the current path for HTA in Egypt. The situational analysis of the HTA system in Egypt provides an overview of the key efforts being undertaken by different health system partners to contribute to achieving UHC in Egypt. It has also highlighted challenges and potential opportunities for the implementation of HTA that should be taken into account when considering the next steps.

The analysis of this small sample of stakeholders reveals that there is basic knowledge and understanding of the current HTA process in Egypt among them. They all are keen to support HTA process implementation in Egypt. Some stakeholders have already taken initiatives to support the HTA process at UPA by establishing HTA units in their organization and showing a willingness to collaborate in the process by providing essential data needed for HTA. This shows that there is a national-level effort to promote HTA and evidence-based decision making in Egypt. However, stakeholders have specified a number of conditions that should be met for them to support HTA efforts. These include aspects such as transparency, input into decision making, evidence-based, timeliness, facilitating market access, and capacity and adequate funding support. These are factors important to stakeholders in Egypt and will need to be carefully considered as Egypt moves forward with the adoption and implementation of HTA. Therefore, the HTA process currently being developed in Egypt should follow a series of principles to be abided by all involved, including the UPA, the UHIA and the different committees. Importantly, some of these aspects will require trade-offs between competing principles. This means that the process should explore how each of the principles is embedded and how they will be dealt with in the process, as well as any plans to review them as HTA matures in Egypt. This will be important, so stakeholders are clear on the expectations from HTA in a medium and longer team and their support for HTA is maintained.

In terms of ensuring adequate capacity and funding to support HTA, the main barrier highlighted by stakeholders is lack of trained staff. They noted that there is a need for capability building for producing and supporting the analyses required for HTA. Capability issues will need to be addressed for successful HTA implementation in Egypt. For example, it will be important that HTA staff within the UPA team, as well as the wider stakeholders involved in the HTA process (such as those part of the scientific committee) are continuously trained to analyze and critically appraise the evidence submissions from the pharmaceutical industry, and that academic support is brought up where needed to supplement the efforts from the UPA.

Stakeholders also showed interest in the output of the HTA process at UPA as it matches their organization’s interests which include resource allocation and maximizing patient benefits. They all expressed the importance of HTA in Egypt being influential and informing decision-making transparently and based on evidence. Having methods and processes for HTA that reflect best practices and are publicly available will facilitate the implementation of these principles in the process.

The results of this study, particularly in terms of the needs for capacity building, align with results from other studies that have assessed the preferred status of implementation of HTA in low- and middle-income countries. For example, the analysis of Fasseeh et al. (Fasseeh et al., 2020) looking at HTA implementation in 11 countries in the MENA region also highlights the limited capacity available for HTA in the region based on few current options for HTA training being available. Limited funding for HTA assessment was also indicated, with private funding having a prominent role, in line with our results. The fact that HTA was perceived not to have a formal role in many of the countries analyzed contrast with our findings where the majority of the stakeholders believe HTA to have a formal role. This difference may be as a result of our analysis being conducted specifically for Egypt, which is currently devising the implementation of HTA following its commitment in legislation, and because our analysis targets a specific group of stakeholders aimed to be involved in the HTA process. The study from Fasseeh et al. also notes that stakeholders in the MENA region seem to have shown their willingness to contribute to HTA by investing in the generation of evidence, such as patient registries, and that transparency is found to be a key factor in legitimizing HTA, in line with the results from our analysis. These results also resonate with analyses conducted in other countries beyond the MENA region, particularly in the reference to the need for further local capacity building such as in the case of Sub-Saharan Africa, Ghana, Indonesia, Nigeria or India. (Downey et al., 2020; Sharma et al., 2020; Uzochukwu et al., 2020; Hollingworth et al., 2021).

This study has strengths and limitations. In terms of limitations, these include: 1) The literature search was based on a non-systematic literature review and was strongly guided by documentation and references shared by the UPA during the scoping meeting. This was mitigated by a separate review of the literature including grey literature, particularly from searches in websites from international organizations such as the World Bank, the WHO or ISPOR. 2) We acknowledge the challenges for conducting an activity like this survey including difficulties around the identification of relevant organizations, willingness to take part, missing data, or complexities in the analysis and generalizability of the results. This hampered our ability to formally analyze the HTA capacity section of the survey. 3) The list of stakeholders was based on a small convenience list of organizations that the UPA had relationships with and were involved with their work. This may have excluded some organizations who do not have a formal relationship with the UPA, and thus prevented taking a broader overview and reduced the ability to assess wider awareness of HTA in Egypt. Furthermore, two key stakeholder groups missing from the questionnaire were patient organizations and medical societies. This means that results should be considered with caution when trying to generalize them. However, the level of seniority of the participants in the survey ratifies the relevance of the results for the purpose of the survey, providing insights from the key organizations that are to be engaged in the HTA process being devised by the UPA in Egypt. That is why the results shown by this survey, albeit based on a small list of organizations, are of relevance to highlight the initial perspectives of stakeholders expected to be directly engaged with or part of the HTA process in Egypt. It would be important to repeat this exercise to explore changes in perceptions and wider involvement of stakeholders in the future. Buy-in from medical societies and patient organizations is crucial in the HTA process and for implementation of guidance in the future. Stronger efforts should be made in the future to approach such important stakeholder groups for HTA. It would also be important to accompany the measuring of the changes in awareness and consensus levels with further stakeholder engagement and progress in the HTA implementation process.

Despite these limitations, the situational analysis including the stakeholder and HTA capacity survey has highlighted enlightening information and allowed for a better understanding of the current state of HTA in Egypt, the relevant organizations associated with HTA in Egypt, their commitment to support HTA efforts, the HTA capacity available and the capability needs in the future.

One of the outputs of this exercise has also been the agreement for different organizations to form a HTA forum, where activities and topics relevant to HTA as well as progress in the process for HTA institutionalization can be discussed, showing the benefits of stakeholder engagement to advance in HTA.

## Conclusions and future research orientations

Using the Framework for institutionalizing HTA (Castro and Suharlim, 2020), it seems that currently Egypt is in the Agenda Setting/Policy Formulation stage in the institutionalization process. In progressing, the steps of adoption and implementation should be given sufficient consideration, with the option to adapt or reframe any policy formulations as a result. HTA should be seen as a dynamic and evolving process, where a simpler process is established at the beginning and where complexity is added as the HTA process matures.

The recent legislation in Egypt has led to the creation of different organizations that will interact with each other in the healthcare system following the ongoing healthcare reform. The roles and responsibilities of each of these organizations are also covered in the legislation. However, the particular responsibilities and roles that each organization as a well as the wider stakeholders will play in the HTA process in Egypt are still being defined, alongside governance arrangements to facilitate these collaborations and engagements. These should be outlined before the HTA process is finalized and further work is undertaken. As the next steps, the process for HTA in Egypt should be clearly described and input from stakeholders should be sought when finalizing it. This process should also account for developing horizon scanning and topic selection criteria that would support the planning and resourcing for HTA at an internal level, while also contributing to transparency with stakeholders at an external level. This aspect is essential as HTA capacity will be limited, particularly at the beginning, and there will be a need to set the number of evaluations that UPA can carry out each year. Adapting the process to the capacity available and the different types of technologies to be evaluated could also be considered. A simple and adaptable process that allows for showing the benefits of HTA to all stakeholders could be considered, including adaptive HTA approaches (Nemzoff et al., 2021; Ollendorf and Pinilla Domínguez, 2021), where relevant. Agreeing on a methodology to be used in HTA would also be important, so stakeholders can input into the process providing the necessary evidence. The value dossier being developed by the UPA in collaboration with the UHIA should be reflective of the methods for HTA in Egypt.

Based on the lack of technical and institutional capability available in Egypt, further training and support on this matter for health system partners should also be considered. These could be accompanied by practical HTA pilots.

There seems to be momentum in Egypt to proceed and advance with HTA institutionalization in the country. It would be important that next steps are considered, build on the skills and capabilities already in place in Egypt, ensure methods and processes are in place and up to date and involve the wider HTA system in Egypt so stakeholders can appropriately contribute and participate in the HTA process. For further wider national-oriented analysis, a larger sample size of participants based on a more inclusive selection process and targeting more organizations and participants should be conducted in the future.

## Data Availability

The raw data supporting the conclusions of this article will be made available by the authors, without undue reservation.
