# Supplementary material for: Institutionalizing health technology assessment in Egypt: Situational analysis and roadmap
Source: Front Pharmacol. 2022 Nov 9;13:1014658. doi: 10.3389/fphar.2022.1014658 (PMC9682258; doi:10.3389/fphar.2022.1014658)
Supplement: Supplementary file 1 [file Table1.DOCX]

# Supplementary Table 1: Targeted literature review search strategy

| Database: Ovid MEDLINE(R) <1946 to August 23, 2021> |
| --- |
| Search Strategy: |
| -------------------------------------------------------------------------------- |
| 1 andorra/ or afghanistan/ or africa, central/ or africa, eastern/ or "africa south of the sahara"/ or africa, southern/ or africa, western/ or albania/ or angola/ or "antigua and barbuda"/ or argentina/ or armenia/ or asia/ or australasia/ or exp australia/ or austria/ or azerbaijan/ or bahamas/ or baltic states/ or bangladesh/ or barbados/ or belgium/ or belize/ or benin/ or bhutan/ or bolivia/ or borneo/ or "bosnia and herzegovina"/ or botswana/ or brazil/ or bulgaria/ or burkina faso/ or burundi/ or cabo verde/ or cambodia/ or cameroon/ or exp canada/ or central african republic/ or chad/ or chile/ or exp china/ or colombia/ or comoros/ or congo/ or costa rica/ or cote d'ivoire/ or croatia/ or cuba/ or czech republic/ or "democratic republic of the congo"/ or exp denmark/ or djibouti/ or dominica/ or dominican republic/ or ecuador/ or el salvador/ or equatorial guinea/ or eritrea/ or estonia/ or eswatini/ or ethiopia/ or europe/ or fiji/ or finland/ or exp france/ or gabon/ or gambia/ or "georgia (republic)"/ or exp germany/ or ghana/ or greece/ or grenada/ or guatemala/ or guinea/ or guinea-bissau/ or guyana/ or haiti/ or honduras/ or hungary/ or iceland/ or independent state of samoa/ or exp india/ or indian ocean islands/ or indochina/ or indonesia/ or ireland/ or exp italy/ or jamaica/ or exp japan/ or kazakhstan/ or kenya/ or korea/ or kosovo/ or kyrgyzstan/ or laos/ or latin america/ or latvia/ or lesotho/ or liberia/ or liechtenstein/ or lithuania/ or luxembourg/ or madagascar/ or malaysia/ or malawi/ or mali/ or malta/ or mauritania/ or mauritius/ or mekong valley/ or mexico/ or melanesia/ or micronesia/ or monaco/ or mongolia/ or montenegro/ or mozambique/ or myanmar/ or namibia/ or nepal/ or netherlands/ or new zealand/ or nicaragua/ or niger/ or nigeria/ or north america/ or exp norway/ or pakistan/ or palau/ or exp panama/ or papua new guinea/ or paraguay/ or peru/ or philippines/ or poland/ or portugal/ or "republic of belarus"/ or exp "republic of korea"/ or "republic of north macedonia"/ or romania/ or exp russia/ or rwanda/ or "saint kitts and nevis"/ or saint lucia/ or "saint vincent and the grenadines"/ or "sao tome and principe"/ or "scandinavian and nordic countries"/ or serbia/ or sierra leone/ or senegal/ or seychelles/ or singapore/ or slovakia/ or slovenia/ or somalia/ or south africa/ or spain/ or sri lanka/ or suriname/ or sweden/ or switzerland/ or taiwan/ or tajikistan/ or tanzania/ or thailand/ or timor-leste/ or togo/ or tonga/ or "trinidad and tobago"/ or turkmenistan/ or uganda/ or ukraine/ or exp united kingdom/ or exp united states/ or uruguay/ or uzbekistan/ or vanuatu/ or venezuela/ or vietnam/ or zambia/ or zimbabwe/ (4221291) |
| 2 algeria/ or bahrain/ or cyprus/ or egypt/ or iran/ or iraq/ or israel/ or jordan/ or kuwait/ or lebanon/ or libya/ or middle east/ or morocco/ or africa, northern/ or oman/ or qatar/ or saudi arabia/ or south sudan/ or sudan/ or syria/ or tunisia/ or turkey/ or united arab emirates/ or yemen/ (186051) |
| 3 1 not 2 (4198350) |
| 4 ((guideline* or guidance or HTA* or comparative effectiveness or (technolog* adj2 (assess* or apprais*))) adj2 (develop* or create* or creating or creation or writ* or produc*)).ti. (1548) |
| 5 ((implement* or disseminat* or provid* or provision or shar*) adj3 (guideline* or guidance or HTA* or comparative effectiveness or (technolog* adj2 (assess* or apprais*)))).ti. (2088) |
| 6 ((guideline* or guidance or HTA* or comparative effectiveness or (technolog* adj2 (assess* or apprais*))) adj2 (program* or organisation* or organization* or company or policy or policies or health system* or health service* or nation* or government* or country* or countries)).ti. (2340) |
| 7 ((health* economic* or pricing* or cost*) adj1 (implement* or scheme* or polic* or guid*)).ti. (181) |
| 8 or/4-7 (5955) |
| 9 8 not 3 (2742) |
| 10 limit 9 to english language (2565) |
| 11 animals/ not humans/ (4843810) |
| 12 10 not 11 (2514) |
| 13 limit 12 to yr="2011 -Current" (1376) |
| 14 limit 13 to (comment or letter or news or newspaper article) (184) |
| 15 13 not 14 (1192) |
| 16 algeria/ or bahrain/ or cyprus/ or egypt/ or iran/ or iraq/ or israel/ or jordan/ or kuwait/ or lebanon/ or libya/ or middle east/ or morocco/ or africa, northern/ or oman/ or qatar/ or saudi arabia/ or south sudan/ or sudan/ or syria/ or tunisia/ or turkey/ or united arab emirates/ or yemen/ (186051) |
| 17 (algeria* or bahrain* or cyprus* or egypt* or iran* or iraq* or israel* or jordan* or kuwait* or lebanon* or libya* or mena or middle east* or morocco* or north* africa* or oman* or qatar* or saudi arabia* or south sudan* or sudan* or syria* or tunisia* or turkey* or united arab emirates or uae or yemen*).tw. (205147) |
| 18 16 or 17 (263713) |
| 19 8 and 18 (79) |
| 20 15 or 19 (1224) |
