# Supplementary material for: Institutionalizing health technology assessment in Egypt: Situational analysis and roadmap
Source: Front Pharmacol. 2022 Nov 9;13:1014658. doi: 10.3389/fphar.2022.1014658 (PMC9682258; doi:10.3389/fphar.2022.1014658)
Supplement: Supplementary file 2 [file DataSheet1.PDF]

In order to submit this form, you should open it with Adobe Acrobat Reader.

UPA Stakeholder Questionairre

This stakeholder questionnaire is a tool to support the identification of the types of stakeholders who are likely to have a role in health technology assessment (HTA) in Egypt as well as the capacity available to support HTA in the country. The questions aim to identify stakeholders and their institutional associations and the available capacity.

Your institution has been identified as a **potential important stakeholder for HTA** in Egypt, and we would like to understand better from you your perspective and views on HTA as well as to identify capacity available within and outside of your organization. The questionnaire will assess:

- The level of political commitment for HTA in Egypt
- The potential for Egypt to benefit from explicit HTA processes, and for economies of scale in running HTA processes
- Current and potential future capacity to develop and implement HTA

Name *

First Name Last Name

Title *

Organization *

Email *

example@example.com

Definitions and context

HTA: A multidisciplinary process that uses explicit methods to determine the value of a health technology at different points in its lifecycle. The purpose is to inform decision-making in order to promote an

equitable, efficient, and high-quality health system.

UPA: The Egyptian Authority for Unified Procurement, Medical Supply and the Management of Medical Technology (UPA) was launched under Law act No.151 of 2019 and the executive regulations of the law were issued under the Decision No. 777 of 2020. UPA was established as an integral part of the current healthcare restructuring in Egypt, and the UPA's role is to contribute effectively to the core values of Egypt's 2030 healthcare vision. One of UPA's key roles is to perform HTA in Egypt.

NICE: The National Institute for Health and Care Excellence (NICE) produces recommendations, information and support for the use of health technologies and services in the NHS and in Social Care in England. The quality of its work is respected throughout the world, and it is recognised as a world-leading institution in HTA.

Consent Form

✓ I {name} , {typeA} on behalf of {typeA12} , understand that I am being asked to participate in a questionnaire activity that is developed jointly by the Egyptian Unified Procurement Authority (UPA) and NICE International in the scope of the project ‘establishing health technology assessment (HTA) in Egypt to ensure equitable access to health technologies.

✓ I understand that the questionnaire and any relevant data to the project will not be shared with any third party, and I understand that the results of this project will be shared with NICE International and relevant stakeholders to the project.

✓ I understand that this questionnaire aims to identify stakeholders relevant to HTA process and their institutional associations and the available capacity and the questions will assess the following:

● The level of political commitment for HTA in Egypt
● The potential for Egypt to benefit from explicit HTA processes, and for economies of scale in running HTA processes
● Current and potential future capacity to develop and implement HTA

1. Current HTA system and institutional aspects

1. In Egypt, are you aware of any central organisation(s) undertaking the role of HTA? If yes, please state their name and current status of recommendations from the organisation(s).You may select more than one option.

	Central role in HTA	Name	Role
Department or unit within Ministry of Health	☐		

Department or unit within another public sector body	☐		
Department or unit within another public sector body	☐		
Standalone public sector institution	☐		
NGO (non-governmental organisation)	☐		
Private Sector	☐		
Others	☐		

2.To your knowledge, how do central government health policy decisions influence decision-making at the local level (for instance, within cities or governorates)? Please describe.

3. What is the role of each of these stakeholders in your country's healthcare spending decisions?

	Role
Ministry of Health	
Priority-setting / HTA body (UPA)	
Other central government body	
Government health insurers	
Private health insurers	
Other academic or research institution	
External donors	
NGO (non-governmental organisation)	
Public healthcare providers (e.g., public hospitals)	
Private healthcare providers (e.g., private hospitals)	
Clinicians	

Professional organisations (e.g medical associations)	
Patient and carer organisations	
Pharmaceutical and devices industry	

4. In Egypt, are there any of the following HTA-related products being developed? *

- ☐ Systematic literature reviews and meta-analysis (e.g., Cochrane reviews)
- ☐ Developing or adapting clinical guidelines, including cost-effectiveness and budget impact considerations
- ☐ Evidence-informed clinical quality indicators
- ☐ HTA analyses (e.g., cost-effectiveness analysis, budget impact analysis)
- ☐ Redesigning basic package / essential list
- ☐ Not sure
- ☐

5. Who pays for the development of any of the above HTA-related products in Egypt? You may select more than one option; and please name the institutions where possible:

	Check	Name of institution
Ministry of Health	☐	
Priority-setting / HTA body (UPA)	☐	
Other central government body	☐	
Government health insurers	☐	
Private health insurers	☐	
Public healthcare providers (e.g., public hospitals)	☐	
Private healthcare providers (e.g., private hospitals)	☐	
Others	☐	

6. Is there a formal process for deciding what HTA-related products will be developed? If yes, please describe. *

7. Who establishes the rules and processes that any institution or technical agencies that develop HTA-related products, for example an HTA body, should follow when developing these HTA-related products? *

9. What are the current arrangements for the quality assurance/peer-reviewing of HTA-related products? Please describe. (For example, an HTA agency may have in-house technical staff to quality assure the analyses produced externally by universities.) *

10. To your knowledge, has Egypt adopted or adapted HTA-related processes, products, or decisions from other countries? Please describe. (e.g., adopting the drug reimbursement decision of a neighbouring country with similar socioeconomic circumstances; or adapting an international clinical guideline to local circumstances) *

11. To what extent do you see health policy decisions in your country influencing that in neighbouring or otherwise similar countries (i.e., in social, economic, or political respects)? Please describe. *

2. Stakeholder attitudes to HTA

12. Are there particularly strong, visible Non- Governmental Organisations (NGO)? Please list their name if any. In addition, please outline if these NGOs have any financial or other links to either the pharmaceutical industry or the government? *

13. Are there patient group associations in Egypt? If yes, please state their name. *

14. Which of these categories best describes your opinion on HTA in Egypt?

- ◯ I strongly support it
- ◯ I somewhat support it
- ◯ I do not support nor oppose it
- ◯ I somewhat oppose it
- ◯ I strongly oppose it

3. Stakeholder expectations of HTA

15. How important to you

Ensuring that the money and resources available for health are distributed in the best way to achieve maximum benefits from the money available

	1	2	3	4	5	
Not important	○	○	○	○	○	Very important

Budget control

	1	2	3	4	5	
Not important	○	○	○	○	○	Very important

Equity

	1	2	3	4	5	
Not important	○	○	○	○	○	Very important

improving quality of healthcare

	1	2	3	4	5	
Not important	○	○	○	○	○	Very important

17. What is the level of need for information (0 = no need as information is adequate / 10 = high level of need as information is not available) on each of the aspects listed below that should be included in the HTA evaluation?

Safety

	1	2	3	4	5	
Low	○	○	○	○	○	High

Comment

Efficacy

	1	2	3	4	5	
Low	○	○	○	○	○	High

Comment

Effectiveness

	1	2	3	4	5	
Low	○	○	○	○	○	High

Comment

Cost effectiveness

	1	2	3	4	5	
Low	○	○	○	○	○	High

Comment

Budget impact

	1	2	3	4	5	
Low	○	○	○	○	○	High

Comment

Social/ ethical concerns

	1	2	3	4	5	
Low	○	○	○	○	○	High

Comment

4. HTA capacity

Technical capacity

18. In your organisation, can you provide detail of the following?

	Number	Job status
1. Staff with a qualification in health economics/health econometrics (first degree, masters, or PhD level).		

2. Staff with a qualification in epidemiology /biostatistics (first degree, masters, or PhD level).

3. Staff with medical or pharmacy degrees.

4. Staff with other relevant university level qualifications in policy analysis, public administration, systematic review and meta-analysis, ethical aspects of outcome and cost measurement, impact analysis, governance and system design, knowledge translation and exchange.

5. Staff with training in health economics/health econometrics/budget impact analysis below the level of a university first degree (e.g., Certificated passing levels of non-degree courses, short courses, training workshops).

6. Staff with non-university training in epidemiology / biostatistics.

7. Staff with non-university training in subjects related to clinical evidence synthesis such as systematic literature reviewing or meta-analysis.

8. Staff with non-university training in policy analysis, public administration, systematic review and meta-analysis, ethical aspects of outcome and cost measurement, impact analysis, governance and system design, knowledge translation and exchange.

9. Staff with experience developing or quality assuring HTA-related products.

19. What experience does your institution have in HTA? **Please provide full references of any HTA reports, Government policy documents, publications in peer review journals, and any policy briefs or documents that have been directly influenced by your institutions research.

	Please list any research projects over the past 5 years with dates, grants obtained, granting agency, and number of staff involved.
1. Systematic reviews including developing review questions, literature search, evidence assessment/review and meta analyses.	
2. Cost effectiveness analysis and related methods (e.g., cost- benefit analysis, multi-criteria decision analysis).	
3.Health economics modelling (indicate approach/method).	
4. Budget Impact analysis.	
5. Health systems policy analysis.	

6. Ethics and values in policy decision making.

7. Knowledge transfer, evidence to policy translation.

20. Please define what needs your institution has in any of the HTA-relevant skills outlined above."Needs" can include staff training (Short-term or long-term); specific technical knowledge, capacity that might be brought to bear through collaborative arrangements with other institutions (e.g., joint posts, visiting posts). Please note the form of assistance, if any, which you might need to build this capacity
